# Identification of Epileptic EEG Signals Through TSK Transfer Learning Fuzzy System

**DOI:** 10.3389/fnins.2021.738268

**Published:** 2021-09-10

**Authors:** Zhaoliang Zheng, Xuan Dong, Jian Yao, Leyuan Zhou, Yang Ding, Aiguo Chen

**Affiliations:** ^1^School of Artificial Intelligence and Computer Science, Jiangnan University, Wuxi, China; ^2^Department of Tuberculosis and Respiratory Diseases, Wuhan Jinyintan Hospital, Wuhan, China; ^3^Department of Radiotherapy, Affiliated Hospital, Jiangnan University, Wuxi, China

**Keywords:** epilepsy EEG signals, TSK fuzzy system, transfer learning, knowledge learning, interpretability

## Abstract

We propose a new model to identify epilepsy EEG signals. Some existing intelligent recognition technologies require that the training set and test set have the same distribution when recognizing EEG signals, some only consider reducing the marginal distribution distance of the data while ignoring the intra-class information of data, and some lack of interpretability. To address these deficiencies, we construct a TSK transfer learning fuzzy system (TSK-TL) based on the easy-to-interpret TSK fuzzy system the transfer learning method. The proposed model is interpretable. By using the information contained in the source domain and target domains more effectively, the requirements for data distribution are further relaxed. It realizes the identification of epilepsy EEG signals in data drift scene. The experimental results show that compared with the existing algorithms, TSK-TL has better performance in EEG recognition of epilepsy.

## Introduction

**FIGURE 1:**
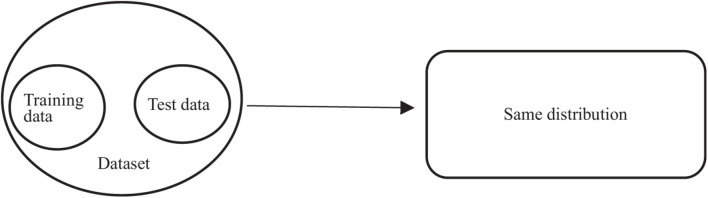
Data distribution scenarios required by traditional methods.

**FIGURE 2:**
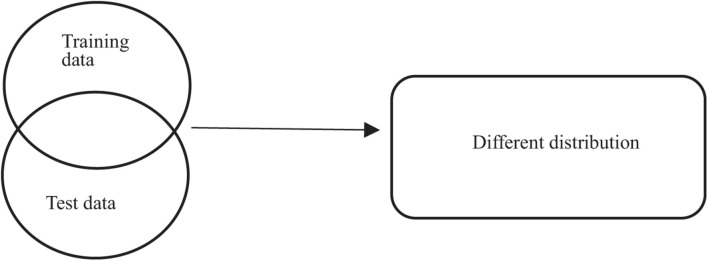
Actual data distribution scenario.

Epilepsy is a disease caused by the sudden discharge of cerebral neurons. EEG technology (Suk et al., 2018) can monitor the changes of brain electrical signals, so we often use EEG intelligent recognition technology to detect epilepsy (Litt et al., 2001; Iasemidis et al., 2003; Dorai and Ponnambalam, 2010). Nowadays, many machines learn algorithms (Wang et al., 2015; Zhang et al., 2021) have been used to recognize epileptic signals, such as Decision Tree (Wang et al., 2015), nearest neighbor (KNN) (Iscan et al., 2011), Naive Bayes Algorithm (NB; Iscan et al., 2011), support vector machines (SVM; Yang et al., 2014), and fuzzy system (Aarabi et al., 2009; Rabbi and Fazel-Rezai, 2012; Deng et al., 2014a, b, 2018; Jiang et al., 2015). It has been proved that these algorithms can detect epilepsy faster and more accurately than doctors. However, as shown in Figure 1, only when the training set and test set obey the similar distribution can they show good classification performance. However, in most cases, as shown in Figure 2, the distribution characteristics of EEG data are not exactly the same. In order to make full use of their similar information, some researchers have proposed to use transfer learning algorithm, such as LMPROJ (Yang et al., 2014) and STL (Wang et al., 2018), applying the old knowledge we have gained to new fields. Different from traditional machine learning, which acquires knowledge from data and applies it to new problems, transfer learning focuses on transferring the learned knowledge to new problems. Although the problem of different data distribution has been solved to a certain extent, these transfer learning algorithms only consider reducing the marginal distribution probability or conditional distribution probability (Deng et al., 2018) of data, without comprehensive balance, and these algorithms lack of interpretability.

To solve these problems, we propose a new method of EEG recognition based on transfer learning and a fuzzy system. The traditional method has a single model structure, so it cannot achieve good results in the face of complex scenes. Different from the traditional method, we pay attention to how to make full use of the previously marked data while ensuring the accuracy of the model on the new task. We not only minimize the marginal distribution or conditional distribution probability, but also combine them to minimize the joint probability distribution (Deng et al., 2018), and reach the best balance between marginal distribution and conditional distribution. In terms of ensuring interpretability, we use the TSK fuzzy system. Its IF-THEN rules can help us understand the rules of model operation more clearly. It has been widely used in data flow modeling, mining tasks, metacognitive learning, and multi-task learning. In order to realize this system, a TSK fuzzy system construction method based on transfer learning (TSK-TL) was developed. The experimental results show that compared with the existing algorithms, TSK-TL has better performance in EEG recognition of epilepsy.

Our contributions are mainly reflected on: (1) The introduction of transfer learning technology (Wang and Mahadevan, 2011; Quanz et al., 2012; Xiao and Guo, 2012), the proposed model in ensuring the accuracy of recognition At the same time, it has higher interpretability; (2) It has stronger robustness and can handle more complex data scenes; (3) It realizes the more accurate identification of epileptic EEG signals in data drift scenarios.

The rest of the manuscript is organized as follows. In the section “Backgrounds,”we briefly introduced the EEG data set, the classical TSK model and the related contents of transfer learning. In section “Identification of Epileptic EEG Signals Through TSK Transfer Learning fuzzysystem,”we first introduced the framework based on transfer learning, and then proposed the objective function of the TSK-TL. In section “Experimental Process and Result Analysis,” we introduce the details of our experiment to test the performance of TSK-TL. The conclusion is given in the last section.

## Backgrounds

This section introduces the data sets and their processing methods used in the research, the classical TSK fuzzy system and the related content of transfer learning.

### Epilepsy EEG Signal Dataset

**FIGURE 3:**
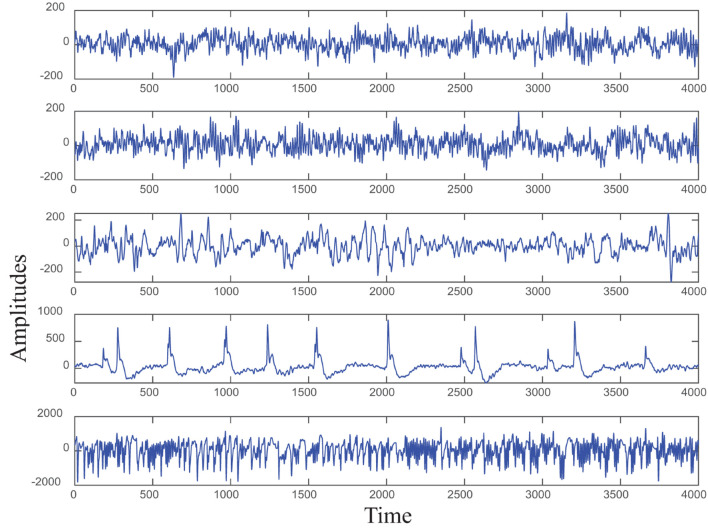
Original epilepsy EEG signals.

The original epileptic EEG data set used in this study is divided into five groups, i.e., Group A to Group E, each group contains 100 single-channel signal segments, and the sampling rate of all samples is adjusted to 173.6 Hz. Among them, the data from healthy people are divided into groups A and B. The difference is that the eyes of group A are opened and group B is closed. The data of groups C, D, and E are obtained from volunteers with epilepsy in different states (Li, 2021). Figure 3 shows the five groups of original epilepsy EEG signals. Table 1 presents these five groups in detail.

**TABLE 1:** Description of the dataset.

Volunteer	Group	Size of group	Description
Healthy volunteer	A	100	EEG signals in healthy volunteers with eyes open
	B	100	EEG signals in healthy volunteers with eyes closed
Volunteer with epilepsy	C	100	EEG signals of the hippocampal structure of the brain during the intermittent period of epilepsy
	D	100	EEG signals in epileptic areas of the brain during intermittent epilepsy
	E	100	EEG signals measured in epileptic patients during the seizure

**FIGURE 4:**
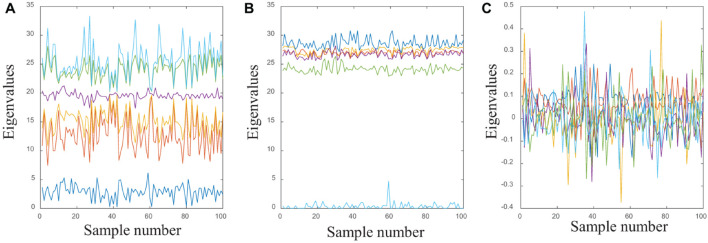
**(A)** The samples of group A processed through WPD; **(B)** the samples of group A processed through STFT; **(C)** the samples of group A processed through KPCA.

The distribution law of EEG signals changes with time. Its amplitude is very small, and it is easy to be affected by other human biological currents such as ECG, EOG, and EMG. At the same time, it has strong randomness, and the noise in the signal is very complicated. Therefore, the experimental results obtained by using the original EEG signal directly are not ideal. According to previous work (Jiang et al., 2017; Tsujikawal et al., 2018), WPD, STFT, and KPCA are three typical feature extraction methods to process epileptic EEG signals (Blanco et al., 1997; Zhang et al., 2000; Srinivasan et al., 2005; Vivaldi and Bassi, 2006; Tzallas et al., 2009; Tang and Durand, 2012; Teng et al., 2017). Figure 4 shows the sample of group A processed by the three feature extraction methods.

### Classical TSK Fuzzy System

Because of its unique interpretability, fuzzy systems has been widely used in modeling and intelligent control. In addition, the output of TSK fuzzy system is more concise. The training process can be transformed into a linear regression problem or a quadratic programming problem, which makes the training process more efficient (Deng et al., 2012; Jiang et al., 2017).

The inference rules of TSK fuzzy system are usually defined as:


(1) $$R^{k}:\text{IF}x_{1}\text{is}A_{1}^{k}\wedge x_{2}\text{is}A_{2}^{k}\wedge \cdots \wedge x_{d}\mathrm{is}A_{d}^{k},$$

$$\text{Then}f^{k}\left(\text{x}\right)=p_{0}^{k}+p_{1}^{k}x_{1}+\cdots +p_{d}^{k}x_{d},k=1,\cdots ,K$$

*K* is the number of fuzzy rules. Each rule is premised on the input vector x = [*x*_1_, *x*_2_, *x*_*d*_]^T^ and maps the fuzzy set in the input space A^*k*^ ⊂ R^*d*^ to the change single case represented by *f*^*k*^(**x**). $A_{i}^{k}$ is the fuzzy subset of the ith dimension of the input vector x under the kth rule. ∧ is a fuzzy conjunction operator. According to previous work (Jiang et al., 2017) the result of the TSK fuzzy model can be expressed as


(2.a) $$y^{0}=\sum_{k=1}^{K}\frac{\mu^{k}\left(x\right)f^{k}\left(x\right)}{\sum_{k^{\prime }=1}^{K}\mu^{k^{\prime }}\left(x\right)}=\sum_{k=1}^{K}{\tilde{\mu }}^{k}\left(x\right)f^{k}\left(x\right)$$

where


(2.b) $$\mu^{k}\left(x\right)=\prod_{i=1}^{d}\mu_{A_{i}^{k}}\left(x_{i}\right)$$

and


(2.c) $${\tilde{\mu }}^{k}\left(x\right)=\mu^{k}\left(x\right)/\sum_{k^{\prime }=1}^{K}\mu^{k^{\prime }}\left(x\right)$$

we use the Gaussian membership function, i.e.,


(2.d) $$\mu_{A_{i}^{k}}\left(x_{i}\right)=\text{exp}\left(\frac{-{\left(x_{i}-c_{i}^{k}\right)}^{2}}{2\delta_{i}^{k}}\right)$$

as the fuzzy membership function. In the paper, we use the FCM algorithm to obtain $c_{i}^{k}$ and $\delta_{i}^{k}$. They can be estimated by the following expressions


(2.e) $$c_{i}^{k}=\sum_{j=1}^{N}u_{jk}x_{ji}/\sum_{j=1}^{N}u_{jk}$$

(2.f) $$\delta_{i}^{k}=h\cdot \sum_{j=1}^{N}u_{jk}{\left(x_{ji}-c_{i}^{k}\right)}^{2}/\sum_{j=1}^{N}u_{jk}$$

*u*_*jk*_ is the fuzzy membership corresponding to the *j*th sample in the *k*th cluster. And *h* is the artificially adjustable scale parameter. After determining these antecedent parameters, let


(3.a) $${\text{x}}_{e}={\left(1,{\text{x}}^{T}\right)}^{T}$$

(3.b) $${\tilde{\text{x}}}^{k}={\tilde{\mu }}^{k}\left(x\right){\text{x}}_{e}$$

(3.c) $${\text{x}}_{g}={\left({\left({\tilde{\text{x}}}^{1}\right)}^{T},{\left({\tilde{\text{x}}}^{2}\right)}^{T},\ldots ,{\left({\tilde{\text{x}}}^{K}\right)}^{T}\right)}^{T}$$

(3.d) $${\text{p}}^{k}={\left(p_{0}^{k},p_{1}^{k},\ldots ,p_{d}^{k}\right)}^{T}$$

(3.e) $${\text{p}}_{g}={\left({\left({\text{p}}^{1}\right)}^{T},{\left({\text{p}}^{2}\right)}^{T},\ldots ,{\left({\text{p}}^{K}\right)}^{T}\right)}^{T}$$

According to the above transformation, Eq. 2a be converted to the following linear regression problem (Jiang et al., 2017).


(3.f) $$y^{o}={\text{p}}_{g}^{T}{\text{x}}_{g}$$

A well-performing algorithm is proposed (Jiang et al., 2017) to train the classic TSK-FS. The objective function of this algorithm is


(4) $$\min_{p_{g}}J_{TSK-FS}\left({\text{P}}_{g}\right)=\frac{1}{2}{\text{P}}_{g}^{T}{\text{P}}_{g}+\frac{{}_{\lambda_{1}}}{2}{||{\text{P}}_{g}^{\text{T}}\text{X}-\text{Y}||}^{2}$$

where $\frac{1}{2}{\text{P}}_{g}^{T}{\text{P}}_{g}$ is the regularization term, which can effectively promote the generalization ability of the TSK-FS; **P**_*g*_ is a consequent parameter; X is the matrix obtained by (5c); _1_ is a regularization parameter. It can adjust the balance between model complexity and error tolerance; [Y = *y*_0_, *y*_1_, …, *y*_*n*_] is the label matrix.

In order to obtain the optimal **P**_*g*_, the derivative of *J*_*TSK*−*FS*_(**P**_*g*_) with respect to **P**_*g*_ can be set to 0, and then the optimal solution of **P**_*g*_ can be obtained as follows:


(5) $${\text{P}}_{g}={\left(\text{I}+\lambda_{1}\cdot {\text{XX}}^{T}\right)}^{-1}\cdot \left(\lambda_{1}\cdot \text{XY}\right)$$

Through the optimal prior and posterior parameters, we can establish a classic TSK fuzzy system.

### Preparatory Knowledge of Transfer Learning

There are three methods for transfer learning: data distribution adaptation, feature selection, and subspace learning (Shi et al., 2013; Zheng, 2021). The basic concept of data distribution adaptation is to make the probability distribution of the data of the source domain (𝒟_*s*_) and the target domain (𝒟_t_) the same or similar through some transformations. Feature selection method considers that the source domain and the target domain contain some common features, and their data distribution is similar. Then, the common features are extracted through the machine learning method, and the model can be built based on these features. The subspace learning method usually assumes that the data of the source domain and target domain will have similar distributions in the transformed subspace, and then learn through statistical feature transformation or manifold transformation.

The joint distribution adaptation (Wang et al., 2018) adopted in the paper belongs to the data distribution adaptive methods. Specifically, the core of joint distribution adaptation is to simultaneously minimize the marginal probability distribution and the conditional probability distribution of the two domains. The distance in machine learning has various forms. Here we use the MMD (Long et al., 2012) as a distance measurement. It can be calculated as follows:


(6) $$MMD_{H}^{2}\left(D_{s},D_{t}\right)={||\frac{1}{n}\sum_{i=1}^{n}\emptyset \left({\text{x}}_{i}\right)-\frac{1}{m}\sum_{j=n+1}^{n+m}\emptyset \left({\text{x}}_{j}\right)||}^{2}$$

where ∅ is a feature mapping, *n* is the number of samples in the source domain and *m* indicates the number of samples in the target domain.

## Identification of Epileptic EEG Signals Through TSK Transfer Learning Fuzzysystem

In this section, we will introduce in detail the transfer learning techniques we use. Combined with the analysis and research on the rules and parameter learning strategies of the classical TSK fuzzy system, a TSK-TL method for detecting epileptic signals is proposed.

### Framework Based on Transfer Learning

**FIGURE 5:**
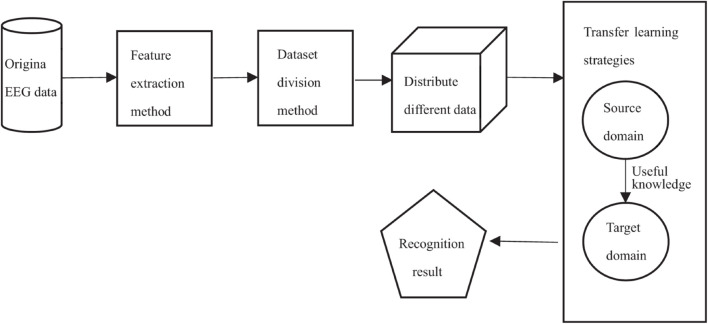
Framework of EEG signal recognition based on transfer learning.

The transfer learning strategy used in this study is divided into two parts: joint distribution adaptation (Zheng, 2021) and historical knowledge learning mechanism. As shown in Figure 5, the framework of epilepsy EEG signals recognition based on transfer learning theory is given. In order to make full use of the information of source domain and target domain, our work mainly includes three steps: (1) minimizing the marginal probability distribution, (2) minimizing the conditional probability distribution, and (3) further learning with historical knowledge.

(1) Minimize the marginal probability distribution

The marginal distributions of the source domain and target domain are represented by *P_s_* and *P_t_*, respectively. The paper builds a new model based on TSK-FS, **P**_*g*_ can be taken as a projected vector, **x**_*g*_ is a projected vector. And in order to make MMD a proper regularization for the classifier, we adopt the projected MMD (Long et al., 2013). Then the marginal probability distribution can be obtained by


(7) $$D\left(P_{s},P_{t}\right)={||\frac{1}{n}\sum_{i=1}^{n}{\text{P}}_{g}^{\text{T}}{\text{x}}_{si}-\frac{1}{m}\sum_{j=1}^{m}{\text{P}}_{g}^{\text{T}}{\text{x}}_{tj}||}^{2}$$

where **x**_*si*_ is the ith sample of the source domain, **x**_*tj*_ is the jth sample of the target domain, *n* and *m* indicate the number of samples in the source domain and target domain, respectively.

(2) Minimize the conditional probability distribution

Reducing the distance of conditional distribution is actually to achieve intra-class migration, but we don’t know the label of the target domain. In the paper, we use some traditional classification algorithms (such as SVM) to obtain the pseudo-label of the target domain. At the same time, we assume that the calculated pseudo-class centroid may be located not far from the real class centroid (Long et al., 2012). Therefore, we can calculate the distance of conditional probability distribution by using both true label and pseudo label. The conditional probability distribution of source domain and target domain are represented by *Q_s_* and *Q_t_*, and then the conditional distribution can be calculated as follows:


(8) $$D^{\left(c\right)}\left(Q_{s},Q_{t}\right)={||\frac{1}{n^{\left(c\right)}}\sum_{X_{i}\in {𝒟}_{s}^{\left(c\right)}}{\text{P}}_{g}^{\text{T}}{\text{x}}_{i}-\frac{1}{m^{\left(c\right)}}\sum_{X_{j}\in {𝒟}_{t}^{\left(c\right)}}{\text{P}}_{g}^{\text{T}}{\text{x}}_{j}||}^{2}$$

where *c* ∈ {0, 1, …, *C*} is the category tag. ${𝒟}_{s}^{\left(c\right)}$ is a set of the data belonging to class c in the source domain and $n^{\left(c\right)}=\left|{𝒟}_{s}^{\left(c\right)}\right|$. Correspondingly, ${𝒟}_{t}^{\left(c\right)}$ is a set of the data belonging to class c in the target domain and $m^{\left(c\right)}=\left|{𝒟}_{t}^{\left(c\right)}\right|$.

By integrating Equations 9 and 10, the joint probability distribution distance can be calculated as follows:


(9) $$D\left(J_{s},J_{t}\right)=D\left(P_{s},P_{t}\right)+\sum_{c=1}^{C}D^{\left(c\right)}\left(Q_{s},Q_{t}\right)$$

(3) Combine the historical knowledge to further learn

The parameter **P**_*g*_0__ obtained through classic TSK-FS is used to further guide the learning, and then the complete tranfer learning item is


(10) $$D\left({𝒟}_{s},{𝒟}_{t}\right)=D\left(J_{s},J_{t}\right)+\lambda_{3}D\left({\text{P}}_{g_{0}},{\text{P}}_{g}\right)$$

where


(11) $$D\left({\text{P}}_{g_{0}},{\text{P}}_{g}\right)={||{\text{P}}_{g_{0}}-{\text{P}}_{g}||}^{2}$$

### The Objective Function of TSK-TL

We design the objective function of TSK-TL as


(12.a) $$\min_{P_{g}}J_{TSK-TL}\left({\text{P}}_{g}\right)=g+\lambda_{2}D\left(J_{s},J_{t}\right)+\lambda_{3}D\left({\text{P}}_{g_{0}},{\text{P}}_{g}\right)$$

where


(12.b) $$g=\frac{1}{2}{\text{P}}_{g}^{T}{\text{P}}_{g}+\frac{\lambda_{1}}{2}{||{\text{P}}_{g}^{\text{T}}{\text{X}}_{s}-\text{Y}||}^{2}$$

$$D\left(J_{s},J_{t}\right)=D\left(P_{s},P_{t}\right)+\sum_{c=1}^{C}D\left(Q_{s},Q_{t}\right)$$

$$={||\frac{1}{n}\sum_{i=1}^{n}{\text{P}}_{g}^{\text{T}}{\text{x}}_{si}-\frac{1}{m}\sum_{j=1}^{m}{\text{P}}_{g}^{\text{T}}{\text{x}}_{tj}||}^{2}$$

(12.c) $$+{||\frac{1}{n^{\left(c\right)}}\sum_{X_{i}\in D_{s}^{\left(c\right)}}{\text{P}}_{g}^{\text{T}}{\text{x}}_{i}-\frac{1}{m^{\left(c\right)}}\sum_{X_{j}\in D_{t}^{\left(c\right)}}{\text{P}}_{g}^{\text{T}}{\text{x}}_{j}||}^{2}$$

(12.d) $$D\left({\text{P}}_{g_{0}},{\text{P}}_{g}\right)={||{\text{P}}_{g_{0}}-{\text{P}}_{g}||}^{2}$$

where **P**_*g*_ is the expected projection of TSK-TL, **P**_*g*_0__ is the consequent parameter of the classical TSK model. **X**_*s*_ is a data matrix from the source domain. **Y** = [*y*_0_, *y*_1_, …, *y*_*n*_] is the label matrix, if the ith sample belongs to healthy volunteers, then *y_i_* is 1, otherwise *y_i_* is -1. And _1, 2, 3_ are the regularization parameters.

Then we further explain the above formula as follows:

(1) Equation 12b is the training model of the classic TSK fuzzy system, so the TSK-TL we proposed inherits all its advantages.
(2) When experimenting with the classical TSK fuzzy system, the result is poor because of the distribution difference of data. By Equation 12c, the joint probability distribution distance is minimized to optimize the experimental results.
(3) In Equation 12d, **P**_*g*_ is further optimized by measuring the distance between **P**_*g*_0__ and **P**_*g*_. If _3_ is infinite and the term is optimal, then **P**_*g*_0__ and **P**_*g*_ are equal.
(4) The regularization parameters _1_ 0, _2_ 0, and _3_ 0 are used to control the balance between different terms. We use the grid search method to determine their values.

### Solution of TSK-TL

In Equation 12c, the first term can be converted as follows:


$$\begin{array}{rcl} & & {\left||\frac{1}{n}\sum_{i=1}^{n}P_{g}^{T}x_{si}-\frac{1}{m}\sum_{j=1}^{m}P_{g}^{T}x_{tj}|\right|}^{2}\\ & & =||\frac{1}{n}P_{g}^{T}{\left[x_{s1}\;x_{s2}\;\cdots \;x_{sn}\right]}_{1^{*}n}{\left[\begin{array}{c} 1\\ 1\\ \vdots \\ 1 \end{array}\right]}_{n^{*}1}\\ & & {\;-\frac{1}{m}P_{g}^{T}{\left[x_{t1}\;x_{t2}\cdots x_{tn}\right]}_{1^{*}m}{\left[\begin{array}{c} 1\\ 1\\ \vdots \\ 1 \end{array}\right]}_{m^{*}1}||}^{2}\\ & & =tr(\frac{1}{n^{2}}P_{g}^{T}X_{s}1{\left(P_{g}^{T}X_{s}1\right)}^{T}+\frac{1}{m^{2}}P_{g}^{T}X_{t}1{\left(P_{g}^{T}X_{t}1\right)}^{T}\\ & & \;-\frac{1}{nm}P_{g}^{T}X_{s}1{\left(P_{g}^{T}X_{s}1\right)}^{T}-\frac{1}{nm}P_{g}^{T}X_{t}1{\left(P_{g}^{T}X_{t}1\right)}^{T})\\ & & \;+tr[P_{g}^{T}(\frac{1}{n^{2}}11^{T}X_{s}^{T}X_{s}+\frac{1}{m^{2}}11^{T}X_{t}^{T}X_{t}\\ & & \;-\frac{1}{nm}11^{T}X_{s}^{T}X_{t}-\frac{1}{nm}11^{T}X_{t}^{T}X_{s})P_{g}]\\ & & \;=tr\left(P_{g}^{T}XM_{0}X^{T}P_{g}\right) \end{array}$$

Similarly, the second term can be converted as:


$$\text{tr}\left({\text{P}}_{g}^{\text{T}}{\text{XM}}_{\text{c}}{\text{X}}^{\text{T}}{\text{P}}_{g}\right)$$

where **X** is a matrix composed of the source domain and target domain data. **M**_c_ is MMD matrix computed as:


(13) $${\left({\text{M}}_{\text{c}}\right)}_{\text{ij}}=\left\{ \begin{array}{c} \frac{1}{n^{\left(c\right)}n^{\left(c\right)}},{\text{x}}_{i},{\text{x}}_{j}\in {𝒟}_{s}^{\left(c\right)}\\ \frac{1}{m^{\left(c\right)}m^{\left(c\right)}},{\text{x}}_{i},{\text{x}}_{j}\in {𝒟}_{t}^{\left(c\right)}\\ \frac{-1}{n^{\left(c\right)}m^{\left(c\right)}},\left\{ \begin{array}{c} {\text{x}}_{i}\in {𝒟}_{s}^{\left(c\right)},{\text{x}}_{j}\in {𝒟}_{t}^{\left(c\right)}\\ {\text{x}}_{j}\in {𝒟}_{s}^{\left(c\right)},{\text{x}}_{i}\in {𝒟}_{t}^{\left(c\right)} \end{array}\right.\\ 0,\text{otherwise} \end{array}\right.$$

So


$$D\left(J_{s},J_{t}\right)=\mathrm{tr}\left({\text{P}}_{g}^{\text{T}}{\text{XMX}}^{\text{T}}{\text{P}}_{g}\right)\text{M}=\sum_{c=0}^{\text{C}}{\text{M}}_{\text{c}}$$

Then,


$$\begin{array}{c} \min_{P_{g}}J_{TSK-TL}\left({\text{P}}_{g}\right)=\frac{1}{2}{\text{P}}_{g}^{T}{\text{P}}_{g}+\frac{\lambda_{1}}{2}{||{\text{P}}_{g}^{\text{T}}{\text{X}}_{s}-\text{Y}||}^{2}\\ \;+\lambda_{2}\text{tr}\left({\text{P}}_{g}^{\text{T}}{\text{XMX}}^{\text{T}}{\text{P}}_{g}\right)+\lambda_{3}{||{\text{P}}_{g_{0}}-{\text{P}}_{g}||}^{2} \end{array}$$

By setting the derivative of *J*_*TSK–TL*_ with respect to **P**_*g*_ to zero, i.e., $\frac{\partial J_{TSK-TL}}{P_{g}}=0$, we can get the optimal solution of **P**_*g*_ as follows:


(14) Pg=((XM+XMT)2XT+λ1XsXsT+λ3I)-1(λ1XsYT+λ3Pg0)

By (7), (15), and (16), we can obtain the optimal posterior parameter **P**_*g*_. Then based on **P**_*g*_, the final decision function can be obtained as follows


(15) $$f\left({\text{x}}_{gi}\right)=\mathrm{sign}\left({\text{P}}_{g}{\text{x}}_{gi}\right)$$

The details of the proposed TSK-TL algorithm are as follows:

**Table:** 

Algorithm of TSK-TL.
***Initialization:*** Set the number of fuzzy rules *K*, the regularization parameters λ_1_, λ_2_, and λ_3_.
***Stage 1: Construction of datasets for linear regression***
Step 1: Through classical FCM or other partition techniques to divide the input space of training data to determine the premise of TSK-FS.
Step 2: Construct the new training dataset and test dataset through (3a)–(3c).
***Stage 2: Computation of historical knowledge parameter*** **P_g_0__**
Step 3: Obtain the **P**_*g*_0__ by (5).
***Stage 3: Obtain the parameter* P_g_ *of TSK-TL***
Step 4: Compute the MMD matrix by (13).
Step 5: Compute the consequent parameter **P**_*g*_ of TSK-TL through (14).
Step 6: Generate the TSK-TL by (15).

## Experimental Process and Results Analysis

In this section, the proposed TSK-TL method is evaluated by classifying EEG signals of epilepsy patients and healthy people. In addition, a comparative study of five traditional machine learning algorithms and two transfer learning algorithms is carried out. Details of the experiments are as follows.

### Experimental Setup

In this paper, we use three classical feature extraction methods, namely WPD, STFT, and KPCA, to obtain EEG datasets from three different views and perform experiments on them, respectively. In each experiment, we set up 10 experimental datasets, and every dataset is composed of part of the data from two or three different groups in this view. The details of these datasets are shown in Table 2. The structure of this dataset ensures that there is no or only a part of the data comimg from the same group. In short, there is no or only a part of the data in the source domain and the test domain has the same distribution.

**TABLE 2:** Structure of experimental datasets.

Datasets	Source domain	Target domain
D1	Group A 50,Group E 50	Group A 50,Group C 50
D2	Group A 50,Group E 50	Group A 50,Group D 50
D3	Group B 50,Group E 50	Group B 50,Group C 50
D4	Group B 50,Group E 50	Group B 50,Group D 50
D5	Group A 50,Group E 50	Group B 50,Group C 50
D6	Group A 50,Group E 50	Group B 50,Group D 50
D7	Group B 50,Group E 50	Group A 50,Group D 50
D8	Group B 50,Group E 50	Group A 50,Group C 50
D9	Group A 50,Group B 50, Group E 50	Group A 50,Group B 50, Group C 50
D10	Group A 50,Group B 50, Group E 50	Group A 50,Group B 50, Group D 50

During the experiment, the experiment of each experimental dataset is repeated 10 times. And take the average result of 10 times to evaluate the performance. The optimal hyperparameters of each experimental model are determined by the grid search. All the algorithms are implemented using MATLAB on a computer with Intel i5-4590 3.3 GHz CPU, 12 GB of RAM. The details of the experimental setup are shown in Table 3.

**TABLE 3:** Experimental setup.

Methods for comparison	Classical recognition methods	Transfer learning recognition methods
	1. KNN 2. NB 3. SVM 4. C4.5 5. TSK-FS	1. LMPROJ 2. STL **3. TSK-TL**
Data label	1. EEG signals from healthy volunteers are labeled “1” 2. EEG signals from volunteers with epilepsy are labeled “−1”	
Performance evaluation	1. Accuracy: The ratio of the number of test data with correct prediction results to the total number of test data 2. Friedman test and Holm’s post hoc test	
Method-specific settings	1. For TSK-FS, the number of fuzzy rules is taken the set {5,10,15,20,25,30} and the regularization parameter λ_1_ from the set {10^−5^,10^−4^,…,10^4^,10^5^} 2. For the proposed TSK-TL, the number of fuzzy rules is taken from the set {5,10,15,20,25,30}, and the regularization parameters λ_1_, λ_2_ and λ_3_ from the set {10^−5^,10^−4^,⋯,10^4^,10^5^} **Note:** Each parameter is determined by the grid search	

### Recognition Performance

In our experiment, five traditional machine learning algorithms and two transfer learning algorithms, i.e., LMPROJ and STL are used for comparative experiments. The results are shown in Tables 4–6. By observing these results, three conclusions can be drawn as follows.

(1) The proposed TSK-TL method not only reduces the difference of data distribution, but also optimizes the formation of new knowledge parameters through the prior knowledge parameters obtained from the classical TSK-FS model. The results show that our method can improve the accuracy of EEG signal recognition.
(2) Compared with the two transfer learning classifiers LMPROJ and STL, TSK-TL is superior to LMPROJ in recognition of epileptic EEG signals, and its performance is better than or at least comparable to the STL method. STL method uses affinity within the class to transfer knowledge within the class, and then to learn. The LMPROJ method only reduces the marginal distribution of data based on the SVM algorithm.
(3) The TSK-TL method we proposed has a good recognition effect in the three views, and the average recognition accuracy in the WPD view is the highest, which points out the direction for us to select an appropriate feature extraction method in practical applications. At the same time, it can be observed that when processing the D5 and D6 data sets, the recognition accuracy is greatly improved compared with other methods, which further verifies that our proposed method has a better effect in the face of data with large differences in data distribution.

**TABLE 4:** Experimental results of eight classifiers in the WPD view.

EEG datasets		Classifiers							
		KNN	NB	SVM	C4.5	TSK-FS	STL	LMPR-OJ	TSK-TL
D1	Mean (SD)	0.8771 (0.0275)	0.5636 (0.0180)	0.9086 (0.0135)	0.8845 (0.0254)	0.9157 (0.0079)	0.8529 (0.0160)	0.9286 (0.0157)	0.9586 (0.0168)
D2	Mean (SD)	0.8857 (0.0223)	0.6127 (0.0119)	0.9300 (0.0153)	0.8818 (0.0218)	0.9329 (0.0170)	0.8843 (0.0321)	0.9357 (0.0127)	0.9571 (0.0111)
D3	Mean (SD)	0.9623 (0.0113)	0.8373 (0.0385)	0.9571 (0.0160)	0.9645 (0.0104)	0.9614 (0.0069)	0.9789 (0.0038)	0.9773 (0.0049)	0.9971 (0.0049)
D4	Mean (SD)	0.9819 (0.0049)	0.8345 (0.0550)	0.9600 (0.0100)	0.9655 (0.0082)	0.9586 (0.0090)	0.9717 (0.0049)	0.9835 (0.0071)	0.9957 (0.0053)
D5	Mean (SD)	0.7529 (0.0472)	0.2655 (0.0151)	0.5900 (0.0183)	0.7936 (0.0566)	0.7271 (0.0287)	0.8629 (0.0522)	0.6829 (0.0801)	0.9543 (0.0199)
D6	Mean (SD)	0.7286 (0.0313)	0.3164 (0.0234)	0.5943 (0.0140)	0.7745 (0.0591)	0.7571 (0.0298)	0.8457 (0.0544)	0.6000 (0.0870)	0.9329 (0.0359)
D7	Mean (SD)	0.9157 (0.0230)	0.7927 (0.0447)	0.7914 (0.0313)	0.7727 (0.0174)	0.9043 (0.0190)	0.9171 (0.0298)	0.8900 (0.0258)	0.9614 (0.0234)
D8	Mean (SD)	0.8914 (0.0267)	0.8309 (0.0411)	0.7843 (0.0113)	0.8136 (0.0344)	0.8986 (0.0302)	0.9297 (0.0168)	0.8757 (0.0172)	0.9571 (0.0256)
D9	Mean (SD)	0.9143 (0.0254)	0.7152 (0.0233)	0.7439 (0.0101)	0.8994 (0.0212)	0.9217 (0.0112)	0.9367 (0.0159)	0.9286 (0.0171)	0.9714 (0.0120)
D10	Mean (SD)	0.9057 (0.0190)	0.7382 (0.0161)	0.7305 (0.0127)	0.9006 (0.0232)	0.9189 (0.0071)	0.9133 (0.0154)	0.9190 (0.0141)	0.9657 (0.0071)

**TABLE 5:** Experimental results of eight classifiers in the STFT view.

EEG datasets		Classifiers							
		KNN	NB	SVM	C4.5	TSK-FS	STL	LMPROJ	TSK-TL
D1	Mean (SD)	0.6390 (0.0265)	0.5682 (0.0227)	0.9729 (0.0111)	0.8336 (0.0150)	0.8343 (0.1409)	0.8871 (0.0138)	0.8828 (0.0138)	0.9800 (0.0100)
D2	Mean (SD)	0.6575 (0.0341)	0.6309 (0.0187)	0.9429 (0.0137)	0.8373 (0.0142)	0.8368 (0.0548)	0.9014 (0.0107)	0.8771 (0.0143)	0.9857 (0.0079)
D3	Mean (SD)	0.5823 (0.0322)	0.5964 (0.0211)	0.9243 (0.0113)	0.7427 (0.1144)	0.9543 (0.0190)	0.8997 (0.0117)	0.9157 (0.0121)	0.9976 (0.0032)
D4	Mean (SD)	0.6060 (0.0481)	0.6409 (0.0489)	0.9647 (0.0053)	0.6927 (0.0827)	0.9171 (0.0496)	0.9167 (0.0026)	0.9529 (0.0049)	0.9986 (0.0053)
D5	Mean (SD)	0.62300 (0)	0.5473 (0.0233)	0.6029 (0.0150)	0.6727 (0.0236)	0.5943 (0.0812)	0.8014 (0.0324)	0.6314 (0.0564)	0.8583 (0.0676)
D6	Mean (SD)	0.5147 (0.0379)	0.4209 (0.0130)	0.5829 (0.0236)	0.6891 (0.0329)	0.5786 (0.1289)	0.7335 (0.0191)	0.6134 (0.0437)	0.8371 (0.0399)
D7	Mean (SD)	0.6015 (0.0452)	0.5909 (0.0212)	0.8400 (0.0163)	0.7127 (0.0163)	0.9071 (0.0160)	0.9046 (0.0107)	0.9171 (0.0160)	0.9786 (0.0079)
D8	Mean (SD)	0.6390 (0.0335)	0.6145 (0.0273)	0.8629 (0.0221)	0.6455 (0.0281)	0.9171 (0.0206)	0.9217 (0.0121)	0.9083 (0.0163)	0.9657 (0.0111)
D9	Mean (SD)	0.6837 (0.0136)	0.6739 (0.0020)	0.8990 (0.0186)	0.8133 (0.0149)	0.8805 (0.0214)	0.9049 (0.0076)	0.9129 (0.0065)	0.9857 (0.0067)
D10	Mean (SD)	0.7130 (0.0183)	0.7048 (0.0052)	0.9162 (0.0076)	0.7818 (0.0085)	0.8967 (0.0144)	0.9123 (0.0233)	0.9076 (0.0081)	0.9771 (0.0115)

**TABLE 6:** Experimental results of eight classifiers in the KPCA view.

EEG datasets		Classifiers							
		KNN	NB	SVM	C4.5	TSK-FS	STL	LMPROJ	TSK-TL
D1	Mean (SD)	0.8813 (0.0336)	0.7760 (0.0219)	0.8167 (0.0462)	0.8955 (0.0121)	0.8700 (0.0394)	0.8414 (0.0308)	0.7781 (0.0164)	0.9120 (0.0311)
D2	Mean (SD)	0.9050 (0.0225)	0.8940 (0.0477)	0.9067 (0.0321)	0.9789 (0.0083)	0.9557 (0.0336)	0.9214 (0.0090)	0.8940 (0.0351)	0.9820 (0.0130)
D3	Mean (SD)	0.9295 (0.0206)	0.9120 (0.0152)	0.8500 (0.0361)	0.8382 (0.0325)	0.9360 (0.0152)	0.9200 (0.0100)	0.8120 (0.0130)	0.9500 (0.0187)
D4	Mean (SD)	0.9450 (0.0176)	0.9260 (0.0114)	0.8367 (0.0252)	0.9591 (0.0138)	0.9680 (0.0045)	0.9343 (0.0127)	0.8127 (0.0158)	0.9820 (0.0084)
D5	Mean (SD)	0.7034 (0.0526)	0.5560 (0.0195)	0.6067 (0.0115)	0.5945 (0.0543)	0.7240 (0.0658)	0.8217 (0.0492)	0.7020 (0.0327)	0.8289 (0.0430)
D6	Mean (SD)	0.7045 (0.0331)	0.6137 (0.0122)	0.6833 (0.0451)	0.7700 (0.0300)	0.7380 (0.0311)	0.8700 (0.0336)	0.7423 (0.0268)	0.8743 (0.0217)
D7	Mean (SD)	0.8827 (0.0239)	0.8109 (0.0138)	0.8700 (0.0400)	0.9218 (0.0271)	0.8943 (0.0292)	0.9186 (0.0135)	0.8343 (0.0151)	0.9420 (0.0228)
D8	Mean (SD)	0.8945 (0.0331)	0.8064 (0.0234)	0.8467 (0.0058)	0.7682 (0.0282)	0.8780 (0.0277)	0.8471 (0.0275)	0.8000 (0.0200)	0.9200 (0.0158)
D9	Mean (SD)	0.9063 (0.0158)	0.7358 (0.0414)	0.6689 (0.0038)	0.8345 (0.0333)	0.8687 (0.0197)	0.8771 (0.0148)	0.8667 (0.0054)	0.9280 (0.0179)
D10	Mean (SD)	0.9533 (0.0169)	0.7655 (0.0409)	0.6667 (0.0017)	0.9810 (0.0054)	0.9520 (0.0073)	0.9210 (0.0178)	0.9371 (0.0143)	0.9827 (0.0060)

### Statistical Analysis

We evaluated the experimental results from a statistical point of view through the Friedman test (Demšar, 2006; Garcia and Herrera, 2008) and the Holm’s *post hoc* test (Demšar, 2006; Garcia and Herrera, 2008). Friedman test is used to calculate the average ranking of the compared methods and to determine whether the observed difference are statistically significant. We set the significance level of the test to 0.05. If the *p*-value is less than 0.05, the null hypothesis H0 is rejected and we can confirm that there are significant difference. The Holm’s *post hoc* test is then performed to evaluate the statistical differences between the control (i.e., the method that achieves the best Friedman rank) and other methods. The results of the Friedman test are shown in Table 7, and the results of the Holm’s *post hoc* test are shown in Table 8.

**TABLE 7:** Comparison of average performance of TSK-Tl and other seven methods using Friedman test (A = 0.05).

Friedman Test for TSK-TL			

Algorithms	Friedman Rank	*p*-Value	H_0_
**KPCA view**			
KNN	3.8	0.000000006179	Rejected
NB	7.05		
SVM	6.4		
C4.5	4.2		
TSK-FS	3.4		
STL	3.8		
LMPROJ	6.35		
TSK-TL	1		
**STFT view**			
KNN	7	0.00000001016	Rejected
NB	7.8		
SVM	3.6		
C4.5	5.3		
TSK-FS	4.6		
STL	3.2		
LMPROJ	3.5		
TSK-TL	1		
**WPD view**			
KNN	4.5	0.000000004808	Rejected
NB	7.5		
SVM	6.5		
C4.5	5.4		
TSK-FS	4.2		
STL	3.3		
LMPROJ	3.6		
TSK-TL	1		

**TABLE 8:** Holm’s *post hoc* comparison of the results of Friedman procedure of TSK-TL and other seven methods

Holm’s *post hoc* comparison for TSK-TL					

i	Algorithm	Z = (R_0_-R_*t*_)/SE	*p*	Holm = /i	H_0_
**KPCA view**					
7	NB	5.522869	0	0.007142	Rejected
6	SVM	4.929503	0.000001	0.008333	Rejected
5	LMPROJ	4.883859	0.000001	0.01	Rejected
4	C4.5	2.921187	0.003487	0.0125	Rejected
3	KNN	2.556039	0.010587	0.016667	Rejected
2	STL	2.556039	0.010587	0.025	Rejected
1	TSK-FS	2.19089	0.02846	0.05	Rejected
**STFT view**					
7	NB	6.207522	0	0.007142	Rejected
6	KNN	5.477226	0	0.008333	Rejected
5	C4.5	3.925345	0.000087	0.01	Rejected
4	TSK-FS	3.286335	0.001015	0.0125	Rejected
3	SVM	2.373464	0.017622	0.016667	Rejected
2	LMPROJ	2.282177	0.022479	0.025	Rejected
1	STL	2.008316	0.04461	0.05	Rejected
**WPD view**					
7	NB	5.933661	0	0.007142	Rejected
6	SVM	5.02079	0.000001	0.008333	Rejected
5	C4.5	4.016632	0.000059	0.01	Rejected
4	KNN	3.195048	0.001398	0.0125	Rejected
3	TSK-FS	2.921187	0.003487	0.016667	Rejected
2	LMPROJ	2.373464	0.017622	0.025	Rejected
1	STL	2.099603	0.035764	0.05	Rejected

As shown in Table 7, the Friedman test results reveal that the TSK-TL method performs better than the other seven classification methods in classification accuracy. And the results of Holm’s *post hoc* in Table 8 also show that compared with other methods, TSK-TL—TL has better performance. This once again proves that our proposed TSK-TL has achieved better results in the detection of epileptic EEG signals.

### Model Analysis

**FIGURE 6:**
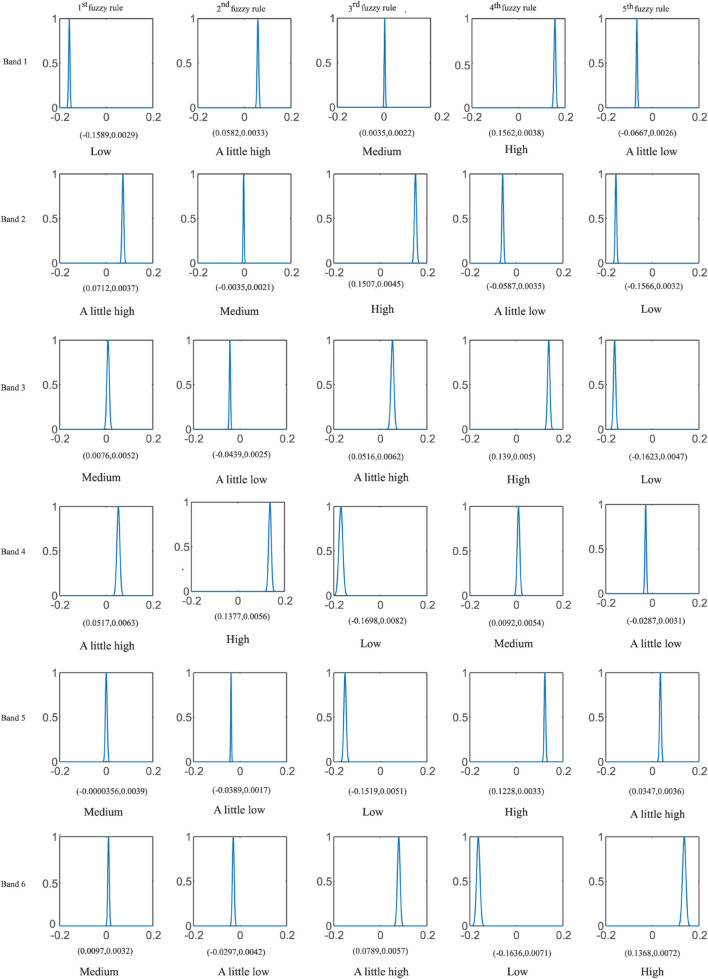
The membership function of each fuzzy rule and the linguistic interpretation of each fuzzy subset of the TSK fuzzy system are obtained from the KPCA view.

In this section, TSK-TL is analyzed through the model trained from the D2 dataset in the KPCA view. Table 9 gives an example of the model, these model parameters prove that TSK-TL inherits the interpretability of the classical TSK fuzzy system. In this example, we set up five fuzzy rules. Figure 6 shows the corresponding MF of each fuzzy set, where each MF has a fuzzy rule description, such as “the energy of a band of EEG signal is Low” (A little low, Medium, A little high or High). Because medical experts in different fields have different interpretations of fuzzy rules, the language description given is only a possible example.

**TABLE 9:** TSK-TL Model with five rules trained on the dataset D2 for the KPCA view.

Fuzzy rules base		

**TSK Fuzzy Rule R^k^: IF$x_{1}isA_{1}^{k}\left(c_{1}^{k},\delta_{1}^{k}\right)⋀x_{2}isA_{2}^{k}\left(c_{2}^{k},\delta_{2}^{k}\right)⋀\cdots ⋀x_{d}isA_{d}^{k}\left(c_{d}^{k},\delta_{d}^{k}\right),Thenf_{\text{x}}\left(x\right)={\text{p}}_{\text{k}}^{0}+{\text{p}}_{\text{k}}^{1}{\text{x}}_{1}+\cdots {\text{p}}_{\text{k}}^{\text{d}}{\text{x}}_{\text{d}}$**		

No. of rules	Antecedent parameters	Consequent parameters
K	${\text{c}}^{\text{k}}={\left(c_{1}^{k},\ldots ,c_{d}^{k}\right)}^{T},\delta^{k}={\left(\delta_{1}^{k},\ldots \delta_{d}^{k}\right)}^{T}$	${\text{P}}_{k}={\left(P_{k}^{0},P_{k}^{1},\ldots ,P_{k}^{d}\right)}^{T}$
1	c^1^ = [−0.1589,0.0712,0.0076,0.0517,−0.000356,0.0097] *δ*^1^ [0.0029,0.0037,0.0052,0.0063,0.0039,0.0032]	p_1_ = [−2.7245,−0.5118,0.9243,−0.295,−0.0378,−0.58,−1.1201]
2	c^2^ = [0.0582,−0.0035,−0.0439,0.1377,−0.0389,−0.0297] *δ*^2^ [0.0033,0.0021,0.0025,0.0056,0.0017,0.0042]	p_2_ = [10.9842,−8.5028,−0.9881,4.7443,−1.8692,3.2596,−3.0533]
3	c^3^ = [0.0035,0.1507,0.0516,−0.1698,−0.1519,0.0789] *δ*^3^ [0.0022,0.0045,0.0062,0.0082,0.0051,0.0057]	p_3_ = [2.045,3.995,−4.3315,−1.4117,−0.9264,−0.0619,0.4859]
4	c^4^ = [0.1562,−0.0587,0.139,0.0092,0.1228,−0.1636] *δ*^4^ [0.0038,0.0035,0.005,0.0054,0.0033,0.0072]	p_4_ = [−3.8877,−2.2364,−0.7276,−1.1165,0.1062,−1.3669,0.9594]
5	c^5^ = [−0.0667,−0.1566,−0.1623,−0.0287,0.0347,0.1368] *delta*^5^ [0.0026,0.0032,0.0047,0.0031,0.0036,0.0071]	p_5_ = [2.0489,4.0366,−4.2784,−1.4602,−1.0065,0.0086,0.9774]

According to the central value from low to high, these five MFs can be expressed as “Low,” “A little low,” “Medium,” “A little high,” and “High”. Finally, through the language expression of the IF-part of the fuzzy rule and the linear function corresponding to the THEN-part of the fuzzy rule, five fuzzy rules of the KPCA view are given:

The first fuzzy rule:

If the energy of EEG signal from band 1 to band 6 is Low, A little high, Medium, A little high, Medium, and Medium, respectively,

THEN the decision value under this rule is obtained by the following formula:


$$\begin{array}{c} f^{1}\left(x\right)=[-2.7245-0.5118x_{1}+0.9243x_{2}-0.295x_{3}\\ \;-0.0378x_{4}-0.58x_{5}-1.1201x_{6}] \end{array}$$

The second fuzzy rule:

If the energy of EEG signal from band 1 to band 6 is A little high, Medium, A little Low, High, A little Low and A little Low, respectively,

THEN the decision value under this rule is obtained by the following formula:


$$\begin{array}{c} f^{2}\left(x\right)=[10.9842-8.5028x_{1}-0.9881x_{2}+4.7443x_{3}\\ \;-1.8692x_{4}+3.2596x_{5}-3.0533x_{6}] \end{array}$$

The third fuzzy rule:

If the energy of EEG signal from band 1 to band 6 is Medium, High, A little high, Low, Low and A little high, respectively,

THEN the decision value under this rule is obtained by the following formula:


$$\begin{array}{c} f^{3}\left(x\right)=[2.045+3.995x_{1}-4.3315x_{2}-1.4117x_{3}\\ \;-0.9264x_{4}-0.0619x_{5}+0.4859x_{6}] \end{array}$$

The fourth fuzzy rule:

If the energy of EEG signal from band 1 to band 6 is High, A little low, High, Medium, High and Low, respectively,

THEN the decision value under this rule is obtained by the following formula:


$$\begin{array}{c} f^{4}\left(x\right)=[-3.8877-2.2364x_{1}-0.7276x_{2}\\ \;-1.1165x_{3}+0.1062x_{4}-1.3669x_{5}0.9594x_{6}] \end{array}$$

The fifth fuzzy rule:

If the energy of EEG signal from band 1 to band 6 is A little Low, Low, Low, A little low, A little high, and High, respectively,

THEN the decision value under this rule is obtained by the following formula:


$$\begin{array}{c} f^{5}\left(x\right)=[2.0489+4.0366x_{1}-4.2784x_{2}-1.4602x_{3}\\ \;-1.0065x_{4}+0.0086x_{5}+0.9774x_{6}] \end{array}$$

According to the final output value, i.e., y = -1 or y = 1, it can be judged whether the patient has epilepsy.

## Conclusion

The study combines the classic TSK fuzzy system with transfer learning technology and proposes a TSK fuzzy system (TSK-TL) that is interpretable and can better adapt to scenarios of data distribution differences. It expands the application scenarios of the model and realizes the recognition of epileptic EEG signals with large data distribution differences in reality.

Although we have proved the effectiveness of TSK-TL, it can be further optimized. For example, there are several predefined parameters in the TSK-TL algorithm. When optimizing them, the optimization process of these parameters takes a lot of time. In the future, we will study the problem and develop more effective algorithms.

## Data Availability Statement

Publicly available datasets were analyzed in this study. This data can be found here: https://github.com/benfulcher/hctsaTutorial_BonnEEG.

## Author Contributions

ZZ developed the theoretical framework and model in this work and drafted the manuscript. XD gave support for medical knowledge. ZZ, XD, JY, and AC implemented the algorithm and performed experiments and result analysis. All authors contributed to the article and approved the submitted version.

## Conflict of Interest

The authors declare that the research was conducted in the absence of any commercial or financial relationships that could be construed as a potential conflict of interest.

## Publisher’s Note

All claims expressed in this article are solely those of the authors and do not necessarily represent those of their affiliated organizations, or those of the publisher, the editors and the reviewers. Any product that may be evaluated in this article, or claim that may be made by its manufacturer, is not guaranteed or endorsed by the publisher.
